# Rapid detection of human cytomegalovirus by multienzyme isothermal rapid amplification and lateral flow dipsticks

**DOI:** 10.3389/fcimb.2024.1430302

**Published:** 2024-07-19

**Authors:** Ming-hui Liu, Xiaochong Guo, Mao-ling Sun, Jia-lun Li, Shu-han Liu, Yun-zhou Chen, Dong-yi Wang, Lan Wang, Yu-zhang Li, Jun Yao, Yang Li, Yu-qing Pan

**Affiliations:** ^1^ Department of Pediatrics, Shengjing Hospital of China Medical University, Shenyang, China; ^2^ School of Forensic Medicine, China Medical University, Shenyang, China; ^3^ Laboratory Animal Center, China Medical University, Shenyang, China; ^4^ Department of Blood Transfusion, Shengjing Hospital of China Medical University, Shenyang, China

**Keywords:** human milk-acquired cytomegalovirus infection, multienzyme isothermal rapid amplification, lateral flow strip, preterm infants, rapid detection

## Abstract

**Introduction:**

Human cytomegalovirus (HCMV) is the most common viral infection seen in newborns. The major route of transmission for acquired human cytomegalovirus infection is breast milk from mothers who are HCMV seropositive to the infants. Thus, a rapid, economical, and simple method to perform HCMV test in breast milk is crucial and necessary for preventing acquired HCMV infection, especially in underdeveloped regions with limited laboratory resources.

**Methods:**

In this study, an effective technique for the detection of HCMV was constructed by combining multienzyme isothermal rapid amplification (MIRA) and lateral flow chromatography strip (LFD). Primers for the conserved HCMV sequence UL83 were utilized for MIRA-LFD testing.

**Results:**

Our results showed that the entire MIRA reaction could be completed in 12 minutes at 37°C, and LFD outcomes could be observed visibly after 10 minutes. The detection sensitivity of this method reached 50 copy/μl. Samples of breast milk were examined to compare MIRA-LFD and conventional qPCR. The accuracy of MIRA-LFD was 100%.

**Discussion:**

The straightforward, rapid, economic features of the test can provide the significant advantages for the prevention of breast milk-acquired cytomegalovirus infection, particularly in resource-limited locations with high seroprevalence of cytomegalovirus.

## 1 Introduction

Human cytomegalovirus (HCMV), a member of the herpes virus Betaherpesvirinae subfamily, is widespread throughout the world. Among the women of reproductive age, the worldwide seroprevalence of cytomegalovirus approximately reaches 86% (Zuhair et al., 2019). One of the most frequent viral causes of congenital infection is HCMV. Although congenital HCMV infection has received increasing attention, the serious consequences of postnatally acquired HCMV infection cannot be disregarded. Breastfeeding is the primary route of postnatally acquired HCMV infection (Hobbs and Davis, 1967; Stagno et al., 1980), and the other routes include blood transfusion and use of blood products (Schleiss, 2006). Term infants have immunity protected by maternal antibodies (Langel et al., 2022), and most postnatally acquired HCMV infections are asymptomatic. However, preterm infants, particularly very-low-birth-weight infants (VLBWI), may suffer catastrophic outcomes from HCMV infection due to their immature immune systems and lacking of protective antibodies from mothers (Perlman and Argyle, 1992). The clinical manifestations of the VLBWI with HCMV infection include sepsis-like syndrome, apnea, bradycardia, neutropenia, thrombocytopenia, hepatosplenomegaly, cholestatic jaundice, HCMV pneumonia, necrotizing enterocolitis, hemorrhagic diarrhea, intestinal stricture, intussusception, etc (Martins-Celini et al., 2016; Garofoli et al., 2021).

Due to a local HCMV reactivation in the mammary gland postpartum, HCMV-seropositive mothers secrete the virus in milk during lactation (Hamprecht et al., 2001). So far, breast milk has been the primary source of postnatally acquired HCMV infection (Bapistella et al., 2019). It is estimated that cumulative transmission rate of HCMV from seropositive mothers to preterm infants is 37% (Hamprecht et al., 2001). However, breast milk is the recommended source of nutrition for infants, and it is also regarded as therapeutic for preterm infants (Quigley et al., 2018). Moreover, it is crucial to initiate milk expression shortly after birth to stimulate milk production and offer early feedings, especially for VLBW infants (Parker et al., 2021). Hence, in order to prevent breast milk-acquired cytomegalovirus infection in premature infants and ensure that the VLBW infants obtain safe breast milk as soon as possible, it is urgently necessary to develop a quick, accurate, and cost-effective technology for detecting the HCMV virus in breast milk.

Currently, there are several methods to detect HCMV, such as virus culture, ELISA (enzyme-linked immunosorbent assay), and PCR. For virus culture, although it is accurate for HCMV identification (Reitter et al., 2016), this method is time-consuming and labor-intensive, which is not suitable for quick detection in the resource-limited area. ELISA has been used for virus detection in breast milk (Drew et al., 2015; Mukhopadhyay et al., 2022). HCMV glycoprotein B binding ELISA was used to detect HCMV immunoglobulin G (IgG) in maternal milk samples. However, a variety of factors blur the accuracy of ELISAs. Nowadays, PCR is the main method for HCMV diagnosis (Nagel et al., 2020). The real-time PCR (RT-PCR) could be effective for detecting HCMV DNA, as it is an accurate tool for deciding whether to eliminate HCMV in breast milk for premature infants (Yasuda et al., 2003), but there are strict requirements for both laboratory staff and equipment. Therefore, popularizing of RT-PCR is difficult and time-consuming in low-resource areas.

Recently, a few feasible and rapid isothermal nucleic detection techniques have been established. Multienzyme isothermal recombinase amplification (MIRA) is a revolutionary nucleic acid amplification technology which is based on recombinase polymerase amplification (RPA) (Sun et al., 2023). Recombinase-Rec A, DNA helicase-gp41, single-stranded binding (SSB) protein, and DNA polymerase I are the four main proteins used in the procedure (Piepenburg et al., 2006). Helicase-GP41 and SSB form the D-Loop and initiate the reaction during amplification. Recombinase and DNA pol I then permit DNA extension at isothermal conditions. The entire reaction can be completed in 5 to 30 minutes at 25~42°C without the use of sophisticated apparatus, which provides a significant advantage over conventional PCR. By hybridizing with labelled colloidal gold, the lateral flow dipstick (LFD) can generate visually observable results in a relatively short period of time (Chen et al., 2021). Furthermore, the utilizing the MIRA-LFD can reduce the expense of virus detection and accelerate the speed of virus detection speed. At the same time, this technology is practical and appropriate for detecting breast milk at home. Therefore, MIRA-LFD detection of HCMV has a potential application prospect in regions with high HCMV seroprevalence and lacking of medical resources.

Therefore, to prevent breast milk-acquired cytomegalovirus infection and ensure the safety of breastfeeding in preterm infants, particularly in resource-limited locations with high seroprevalence of cytomegalovirus, we combined MIRA and LFD to establish a straightforward, rapid and affordable method for HCMV detection.

## 2 Methods

### 2.1 Primer design

The HCMV genome contains UL83(KJ426589.1.), which is a conserved region with a length of 1683 bp (Mengelle et al., 2003; Smithers-Sheedy et al., 2017). Therefore, UL83 region was selected to generate the primers by Premier 5.0 according to the primer design criteria of Amp-Future Biotechnology Co. LTD. (Weifang, China). The primer length ranged from 25 to 35 base pairs with the amplicons ranging from 150 to 300 base pairs (**Table 1**). The specificity of the primers was examined by using BLAST (http://www.ncbi.nlm.nih.gov/blast/Blast.cgi).

**Table 1:** The primers used for MIRA.

Primer	Sequence (5′ to 3′)	Position in HCMV sequence*	Product length(bp)
F-U1	TGGCTGGTGAAGGTGGGGGGCTCGCTGTA	121562-121590	236
R-U1	GTGGCTTTTACCTCACACGAGCATTTTGGGC	121767-121797	
F-U2	CAGCCCAAAATGCTCGTGTGAGGTAAAAG	121765-121793	165
R-U2	GTGGAAGAGGACCTGACGATGACCCG	121904-121929	
F-U1 labeled	[5′-FAM]- TGGCTGGTGAAGGTGGGGGGCTCGCTGTA	121562-121590	236
R-U1 labeled	[5′-biotin]- GTGGCTTTTACCTCACACGAGCATTTTGGGC	121767-121797	

*NCBI Reference Sequence: KJ426589.1.

### 2.2 HCMV template preparation

The MIRA reaction was carried out using the template of plasmid DNA containing HCMV fragments. The HCMV UL83 sequence was cloned into pCDNA3.1 (+) by Qingke Biotechnology Co., LTD. (Nanjing, China). After plasmid extraction, DNA concentration was measured using NanoDrop™ One ultraviolet spectrophotometer (ThermoFisher Scientific, Waltham, Massachusetts, USA) and the copy number was calculated according to Waggoner et al.’s study (Waggoner et al., 2012).

### 2.3 MIRA

The MIRA reaction was performed using the AMP-Future Biotech Co. Ltd. Kit (# WLB8201KIT). In accordance with the manufacturer’s recommendations, the reactions were carried out for 30 minutes in a water bath at 37°C. Two pairs of the primers (First pair: F-U1 and R-U1; Second pair: F-U2 ad R-U2) were designed in order to select the suitable one (**Table 1**). The amplified products (2μl) were electrophoresed on a 2% agarose gel at 110 V for 45 minutes after being purified with phenol. After separation, the products were detected using an automated digital gel image analysis system (Tanon, Shanghai, China). Water and milk from seronegative patients were utilized as negative controls, while the template DNA and primers in the kit was used as a positive control.

### 2.4 MIRA-LFD

FAM and biotin were added as labels to the 5’ ends of the forward and reverse primers, designated F-U1 labelled and R-U1 labelled, respectively (**Table 1**). MIRA-LFD was carried out by an amplification kit (Colloidal gold test strip type) (#WLB8203KIT, AMP-Future Biotech Co. Ltd). The reactions were finished in 12 minutes in a water bath at 37°C, then the products were diluted 1:5000 with H_2_O. The LFD (#JY0201, Baoying Tonghu Biotechnology Co. Ltd., Beijing) was visually examined using aliquots of the diluted products (100 μl) after 10 minutes.

### 2.5 Sensitivity and specificity analysis

The MIRA reaction’s sensitivity was tested by serially diluting HCMV plasmid DNA with ddH_2_O from 5×10^6^ copies/μl to 5×10^3^ copies/μl, and MIRA products were electrophoresed on a 2% agarose gel at 110 V for 45 minutes after being purified with phenol. The dilutions were tested by qPCR and MIRA-LFD in parallel. qPCR was carried out with TaKaRa TB Green Premix Ex Taq II (Tli RNaseH Plus, TaKaRa, Dalian, China) using the Applied Biosystems 7500 Real-Time PCR equipment (ThermoFisher Scientific). The qPCR primers were previously reported (Valdez-Salazar et al., 2020). The reaction conditions were as follows: 95°C for 30 s, 40 cycles at 95°C for 5 s and 60°C for 34 s. HCMV plasmid DNA diluted from 5×10^6^ copies/μl to 5 copies/μl were employed as the templates. Each amplification reaction was repeated three times.

For specificity, the cross reactivity of the MIRA assay was examined for common infectious viruses, including rotavirus, human immunodeficiency virus-1(HIV-1), herpes simplex virus-1(HSV-1), Japanese encephalitis virus (JEV), and hepatitis B and C viruses. All the templates used in specificity analysis were 5×10^8^ plasmid DNA contains the target virus sequence. Additionally, we assessed the MIRA-LFD reaction’s specificity using the human genome as the template (Sun et al., 2023).

### 2.6 DNA sequencing

MIRA products were sequenced using the ABI3730XL (Taihe Biotechnology Co. Ltd, Beijing, China) for Sanger DNA sequencing. Sequencing was carried out using Primer F-U1 and R-U1.

### 2.7 Clinical sample analysis

Written informed consent was obtained from all the subjects. The breast milk samples were obtained from the local community hospital, of which 20 were positively diagnosed as HCMV infection. The DNA extraction was performed from the samples using QIAamp Blood DNA Kit (Qiagen, Germany) and detected by MIRA-LED reaction accordingly.

## 3 Results

### 3.1 Selection of primers

**Figure 1:**
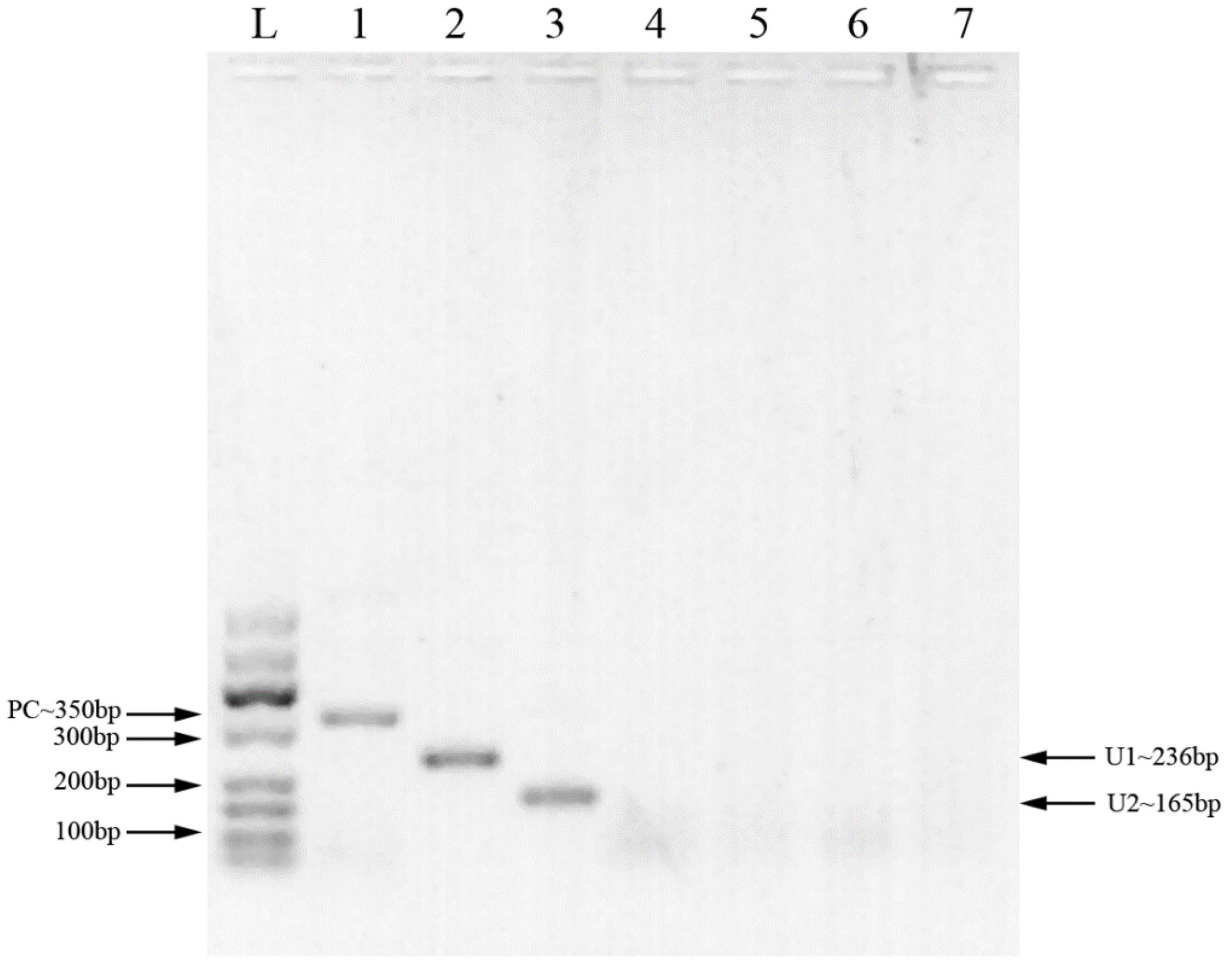
MIRA products detected by 2% agarose gel. L: 50-600bp DNA Ladder; 1: positive control (∼350 bp); 2: primer U1 (∼236 bp); 3: primer U2 (∼165 bp); 4: primer U1, negative control (water); 5: primer U2, negative control (water); 6: primer U1, negative control (milk); 7: primer U2, negative control (milk).

**Figure 2:**
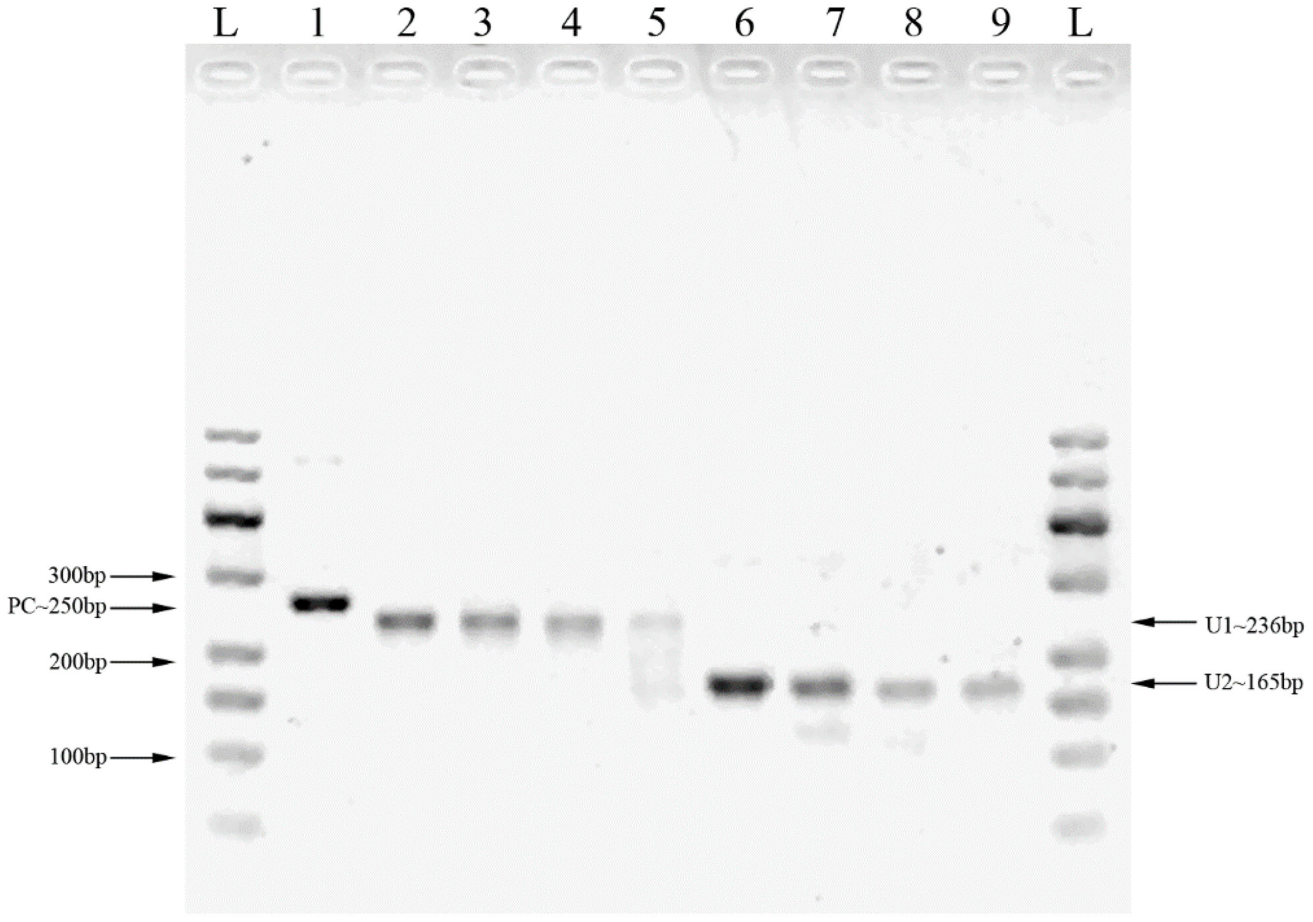
The MIRA products for the serial dilution of HCMV plasmid DNA detected by 2% agarose gel. L: 50-600bp DNA Ladder; 1: positive control (∼250 bp); 2: 5×10^6^ copies/μl template with U1 primers; 3: 5×10^5^ copies/μl template with U1 primers; 4: 5×10^4^ copies/μl template with U1 primers; 5: 5×10^3^ copies/μl template with U1 primers; 6: 5×10^6^ copies/μl template with U2 primers; 7: 5×10^5^ copies/μl template with U2 primers; 8: 5×10^4^ copies/μl template with U2 primers; 9: 5×10^3^ copies/μl template with U2 primers.

**Figure 3:**
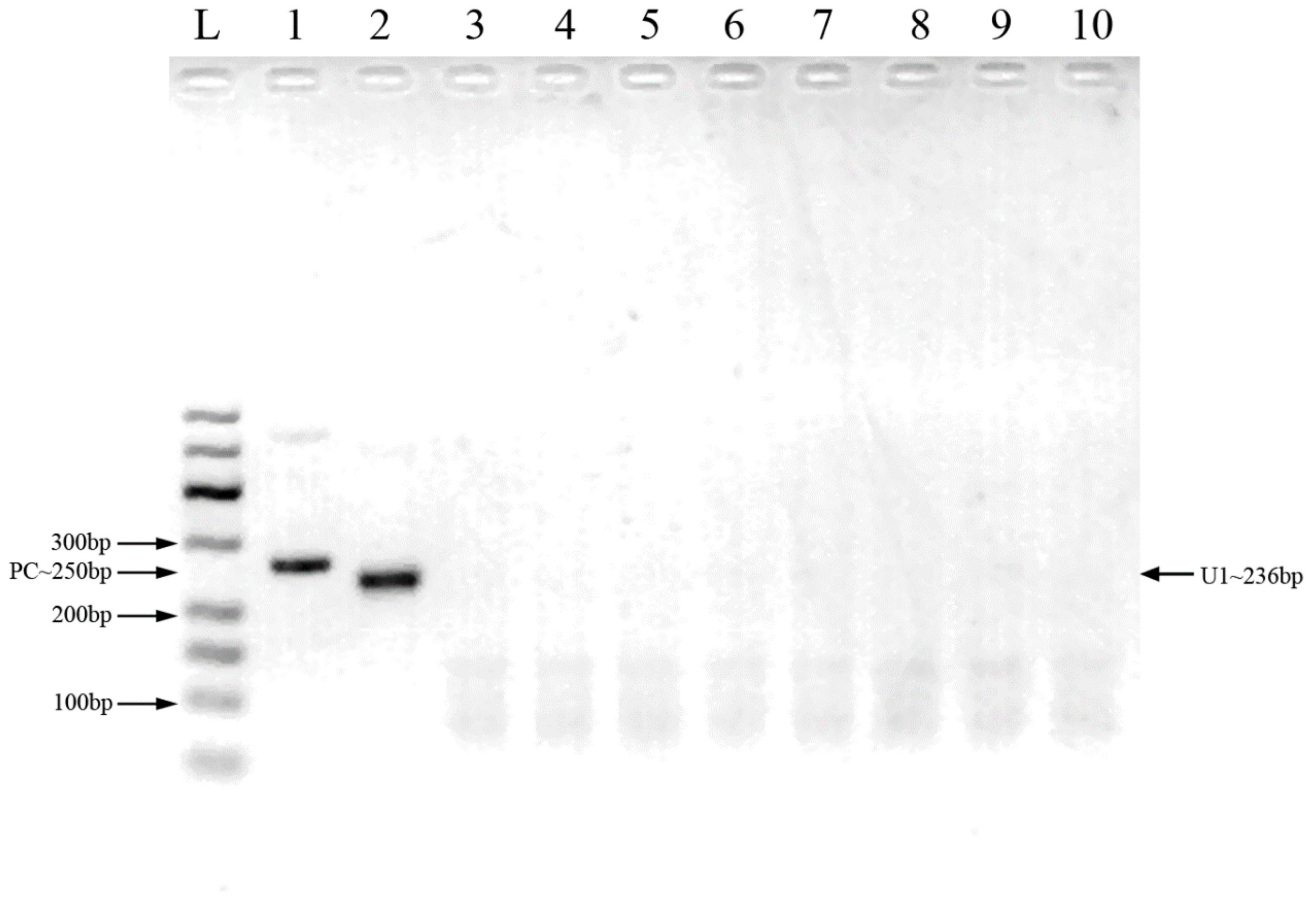
Cross-reactivity testing against multiple pathogens using MIRA. L: 50-600bp DNA Ladder; 1: positive control (∼250 bp); 2: HCMV; 3: HBV; 4: HCV; 5: HEV; 6: HIV-1; 7: HSV-1; 8: JEV; 9: rotavirus 10: human genome.

**Figure 4:**
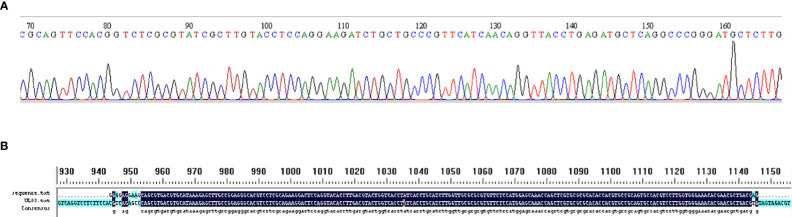
Validation of MIRA by Sanger sequence. **(A)** Sanger sequence of MIRA amplified product using HCMV plasmid template. **(B)** The result of sequence alignment.

Two pairs of primers were designed for HCMV detection (**Table 1**; **Figure 1**). By utilizing MIRA to amplify 10-fold serial dilutions of HCMV plasmid DNA, the optimal primer pair was screened (**Figure 2**). The results demonstrated that while both the U1 and U2 primers were suited to detect templates at concentrations ranging from 5×10^3^ copies/μl to 5×10^6^ copies/μl, the U1 primers were proved to be more efficient and generated more intense bands. Thus, we utilized the U1 primers for the subsequent studies. By applying the U1 primers, MIRA was unable to detect any of the other pathogens (**Figure 3**). The sequence of the MIRA product, which was generated with the U1 primer, was shown (**Figure 4A**). In addition, the result of sequence alignment was shown (**Figure 4B**).

### 3.2 MIRA-LFD

**Figure 5:**
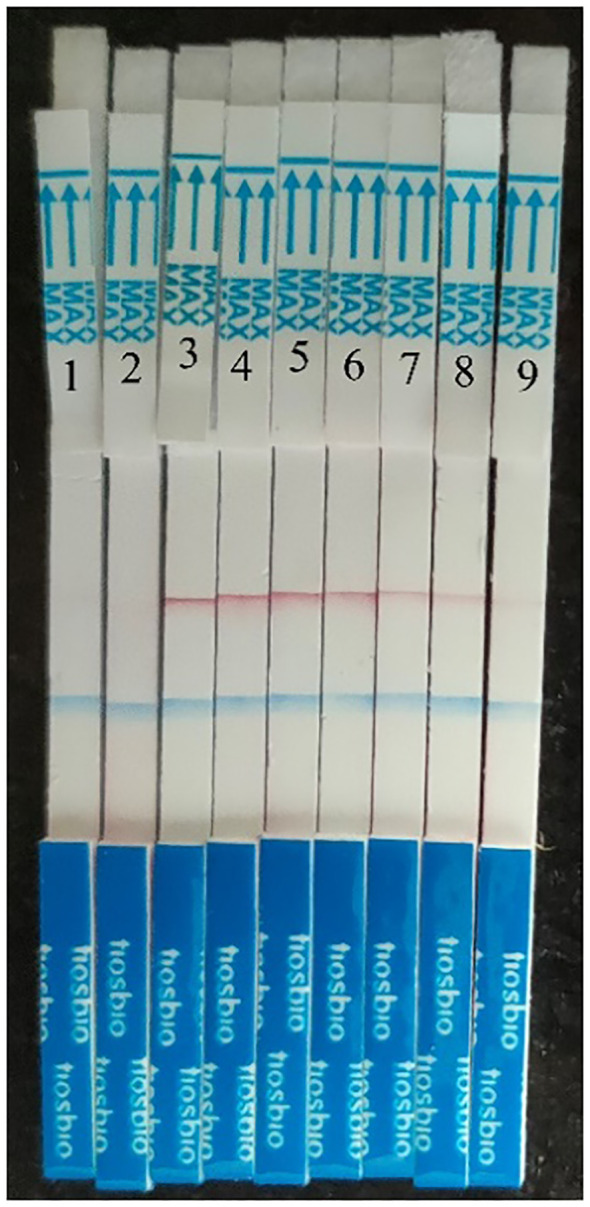
Serial dilution of HCMV plasmid DNA detected by MIRA-LFD. 1: water template; 2: human genome template; 3: 5×10^6^ copies//μl template; 4: 5×10^5^ copies/μl template; 5: 5×10^4^ copies/μl template; 6: 5×10^3^ copies/μl template; 7: 5×10^2^ copies/μl template; 8: 5×10^1^ copies/μl template. 9: 5 copies/μl template.

**Figure 6:**
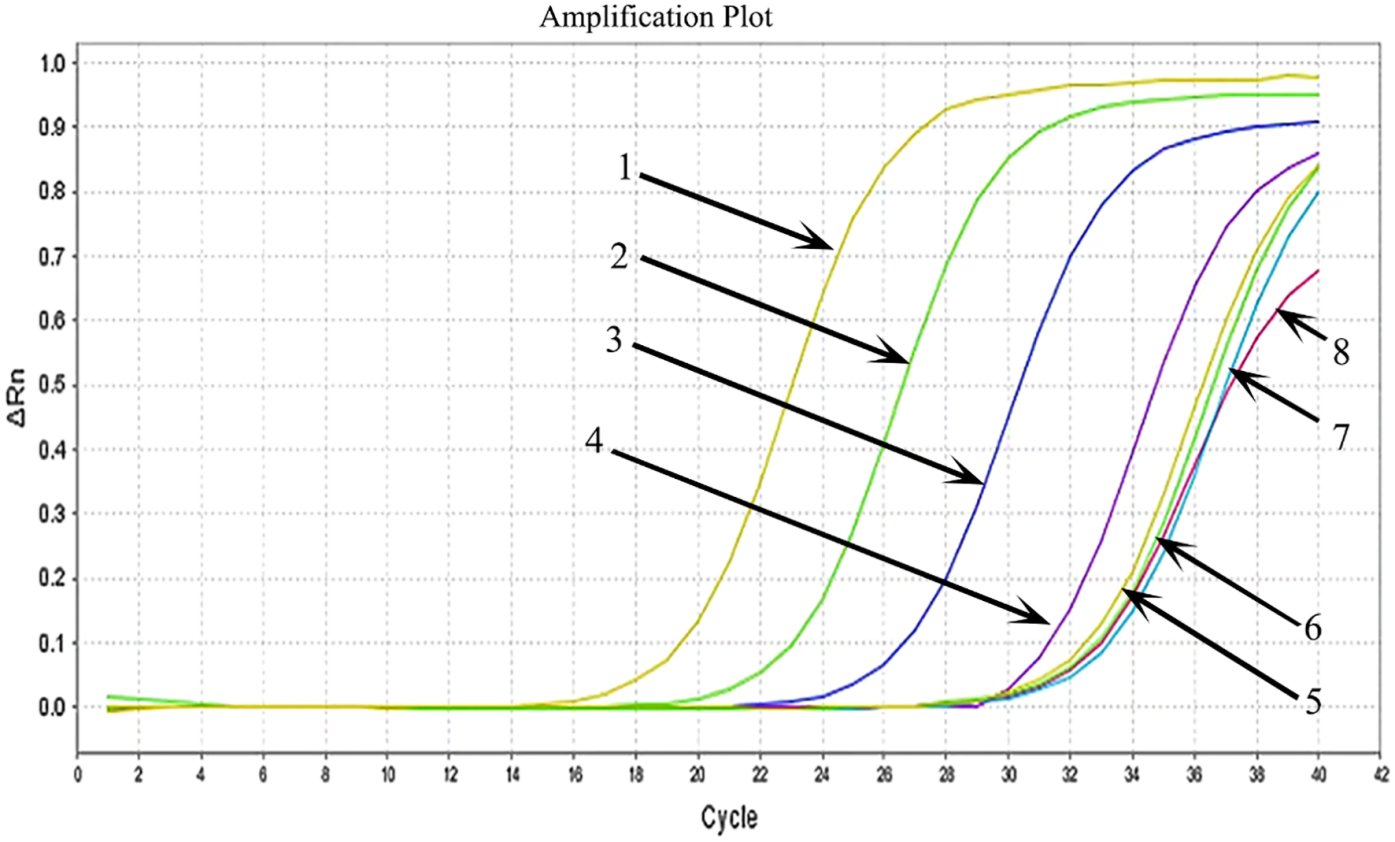
Serial dilution of HCMV plasmid DNA detected by qPCR. 1: 5×10^6^ copies/μl template; 2: 5×10^5^ copies/μl template; 3: 5×10^4^ copies/μl template; 4: 5×10^3^ copies/μl template; 5: 5×10^2^ copies/μl template; 6: 5×10^1^ copies/μl template; 7: 5 copies/μl template; 8: water template.

HCMV plasmid DNA was 10-fold serially diluted (from 5×10^6^ copies/μl to 5 copies/μl), and the samples were then examined using MIRA-LFD (**Figure 5**). We found that the strip could identify plasmid DNA at concentrations as low as 50 copy/μl. In contrast, no amplified fragments were observed for the water and human genome template. In addition, we used qPCR to analyze 10-fold serial dilutions of plasmid from 5×10^6^ copies/μl to 5 copies/μl, which had a detection limit of 5×10^3^ copies/μl (**Figure 6**). The findings demonstrated that MIRA-LFD was more sensitive than qPCR for detecting HCMV.

### 3.3 Clinical sample analysis

**Figure 7:**
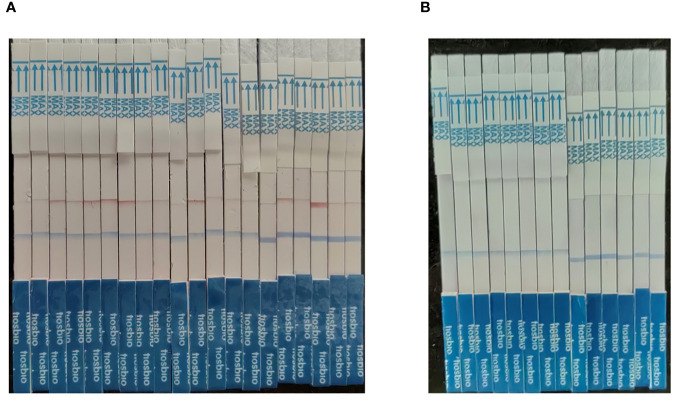
The results of the clinical samples. **(A)** The testing results of 20 positive individuals. **(B)** The testing results of 14 negative individuals.

The MIRA-LFD assay was validated using clinical samples from 20 positive and 14 negative individuals (**Table 2**; **Figure 7**). The MIRA-LFD results were compatible with the results of qPCR, demonstrating that MIRA-LFD had the potential in clinical.

**Table 2:** HCMV detection in clinical samples.

		Real time-PCR			Accuracy rate
		positive	negative	total	
MIRA-LFD	positive	20	0	20	100%
	negative	0	14	14	
	total	20	14	34	

## 4 Discussion

**Figure 8:**
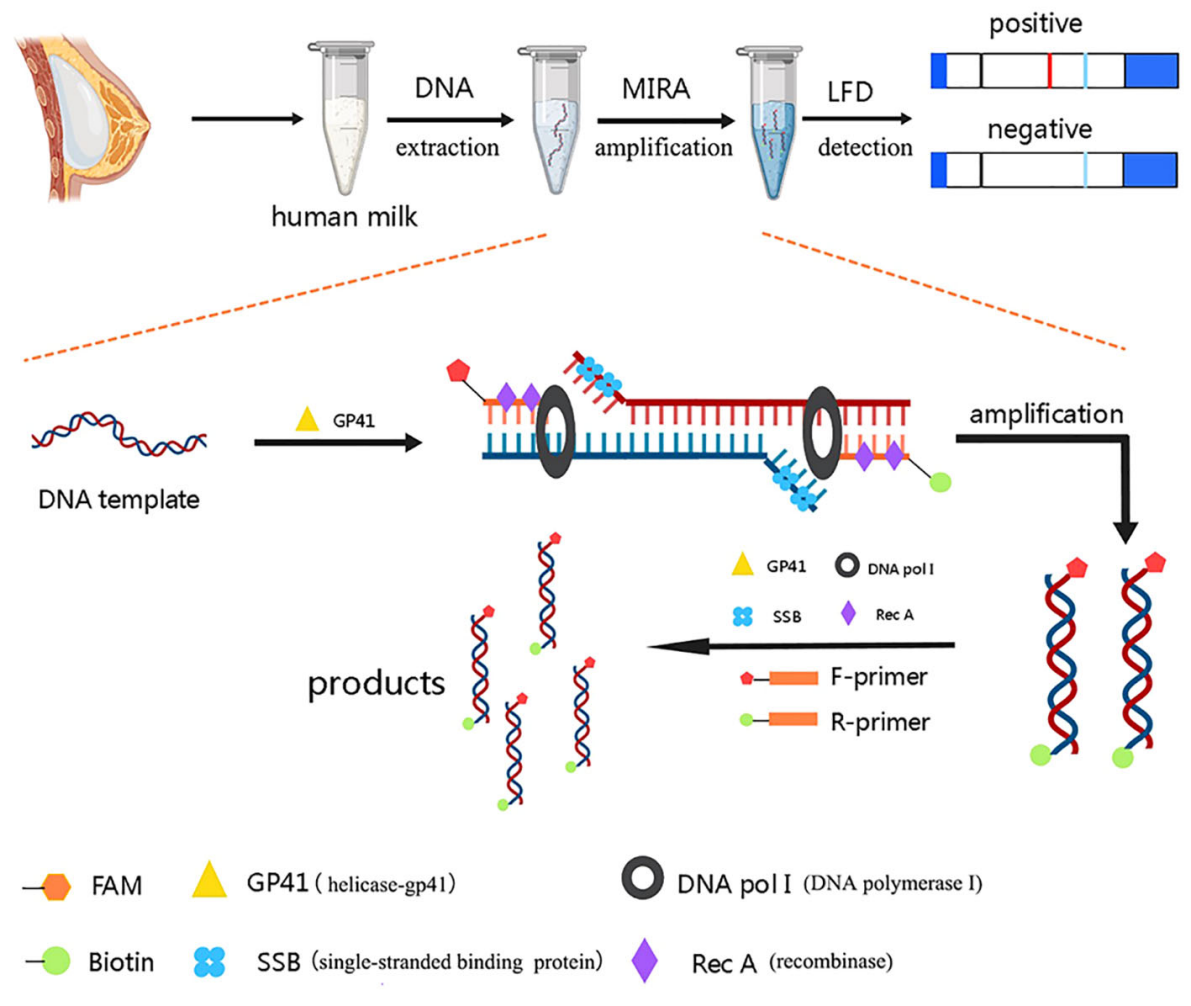
Scheme summarizing of the complete testing process.

A rapid, effective, and economical HCMV detection method can prevent acquired cytomegalovirus infection in premature infants, which decrease the significant cost burden of HCMV-related illnesses. In this study, we combined MIRA with LFD to create a brand-new approach for HCMV detection, which could be appropriate for detection at home. The scheme of the method was summarized in **Figure 8**. HCMV DNA was extracted from breast milk samples by a DNA quick release agent after moderate vortexing and centrifugation. The entire MIRA reaction could be completed in 12 minutes at 37°C. The product was then diluted and measured by the strip, yielding positive results visually observed after 10 min. This method does not require sophisticated apparatus or skilled operators. In conclusion, our study provides an innovative method for rapid HCMV detection, which can be applied in areas with limited medical resources to minimize the serious illness caused by HCMV infection in premature infants. In addition, a rapid detection of viruses in breast milk can hasten the initiation of breastfeeding for VLBWI.

With the rapid development of molecular biology technology, a number of isothermal amplification techniques have been developed to overcome the drawbacks of PCR technology (Deng and Gao, 2015). These include multiple cross displacement amplification (MCDA), loop mediated isothermal amplification (LAMP), cross primer amplification (CPA), strand displacement amplification (SDA), helicase dependent amplification (HDA), recombinant enzyme polymerase amplification (RPA), polymerase spiral response (PSR), and others. For MCDA, LAMP, and CPA (Fang et al., 2009; Li et al., 2019; Sun et al., 2022), the primer design procedure is extremely complicated, and the amplicon validation stage is also challenging. SDA has a low amplification efficiency for long targets and requires sample preparation (Yan et al., 2014; Wang et al., 2022). A significant drawback of HDA is the need for a lengthy optimization period to obtain an optimal equilibrium between the helicase DNA polymerase and reaction parameters (Barreda-García et al., 2018; Botella, 2022). Multiple enzymes are required for RPA reactions, and the conditions are more stringent (James and Macdonald, 2015). PSR is carried out at 65°C for approximately 70 minutes (Jangra et al., 2022). Some PCR-based techniques, including quantitative PCR and fast single tube nested real-time PCR, have been used for detecting breast milk (Romero-Gómez et al., 2015; Martins-Celini et al., 2016). Although the sensitivity and specificity of such methods are favorable, they require sophisticated apparatus and expensive equipment. Thus, it is difficulty to be widely applied in regions that are underdeveloped with limited resources.

Our MIRA method reacts at only 37°C for 12 minutes for the quick detection of HCMV in breast milk, which is convenient and feasible to confirm the safety of premature infants’ breastfeeding. MIRA is also a highly sensitive and selective isothermal amplification technology that requires little sample preparation and can amplify as few as 1 to 10 copies of DNA template in no more than 20 minutes (Lobato and O’Sullivan, 2018). Additionally, it does not require the complicated laboratory conditions of prior techniques (Hoff, 2006). Recombinase, SSB protein, and DNA polymerase cooperate in the reaction system to rapidly amplify nucleic acids at room temperature using HCMV DNA strands as templates (Sun et al., 2021; Sun et al., 2023). After amplification, LFD technology can be used to rapidly generate clear detection results.

The multienzyme isothermal rapid amplification method is almost identical to RPA, both are based on the biological recombination repair mechanism to accomplish isothermal amplification *in vitro*. However, RPA is not ideal for distinguishing mutations or identifying mutations based on nested-RPA. At the same time, the cost of RPA assay is expensive which could reach 12 dollars/reaction. Comparing with RPA, the core functional enzyme system in MIRA is more complete and efficient. In addition, MIRA also has a steadier reaction and a lower cost. The entire detection process can be finished in 30 minutes as opposed to 2 hours for PCR, or several days for virus culture. About $6.50 per sample is spent on the MIRA reaction, which is nearly 50% cheaper than the RPA reaction. Furthermore, rather than requiring sophisticated PCR apparatus, the MIRA reaction just requires a 12-minute reaction at a constant temperature of 37°C. As a result, this technology provides a fast and sensitive method for HCMV identification in milk samples, which can prevent the transmission of HCMV to infants.

There are still several potential limitations in the present study. First, non-specific amplification is produced by MIRA reactions when using the low-concentration templates. It is necessary to optimize virus detection at low concentrations. Furthermore, as aerosol contamination might happen when the tube cap is opened, using LFDs to interpret MIRA results may result in false positive results. In order to prevent the carry-over contamination of viral nucleic acid, a thermostatic amplification integrated detecting equipment need be employed for testing. In our study, only breast milk was examined by MIRA assay. However, blood, saliva, urine, and cervical secretions are also crucial for both congenital and acquired HCMV infection detection (Pesch et al., 2022; Sapuan et al., 2022; Dunn et al., 2023). Thus, more efforts are required to expand the application of MIRA-LFD for screening for congenital cytomegalovirus infection and prevention of acquired HCMV infection.

## 5 Conclusion

In summary, the combination of MIRA and LFD is a straightforward, sensitive cost-effective method without specific equipment. The whole MIRA-LFD procedure only takes 12 min at 37°C, and the results are visible after 10 min using the test strip. Therefore, this approach has significant potential in resource-limited areas, particularly in areas with high HCMV seroprevalence.

## Data availability statement

The original contributions presented in the study are included in the article/supplementary material. Further inquiries can be directed to the corresponding authors.

## Ethics statement

The studies involving humans were approved by Shengjing Hospital of China Medical University. The studies were conducted in accordance with the local legislation and institutional requirements. The participants provided their written informed consent to participate in this study.

## Author contributions

M-HL: Formal analysis, Writing – original draft. XG: Investigation, Writing – review & editing. M-LS: Methodology, Writing – review & editing. J-LL: Methodology, Writing – review & editing. S-HL: Resources, Writing – review & editing. Y-ZC: Resources, Writing – review & editing. DW: Formal analysis, Writing – review & editing. LW: Resources, Validation, Writing – review & editing. Y-ZL: Validation, Writing – review & editing. JY: Writing – review & editing, Writing – original draft. YL: Conceptualization, Data curation, Writing – original draft, Writing – review & editing. Y-QP: Funding acquisition, Supervision, Writing – original draft, Writing – review & editing.
